# Trajectory optimization of UAV-IRS assisted 6G THz network using deep reinforcement learning approach

**DOI:** 10.1038/s41598-024-68459-8

**Published:** 2024-08-09

**Authors:** Amany M. Saleh, Shereen S. Omar, Ahmed M. Abd El-Haleem, Ibrahim I. Ibrahim, Mostafa M. Abdelhakam

**Affiliations:** Electronics and Communications Department, Faculty of Engineering, Helwan University, Cairo, Egypt

**Keywords:** Sixth-generation (6G) network, Terahertz (THz) communication, Unmanned aerial vehicles (UAVs), Intelligent reconfigurable surface (IRS), User grouping design, UAV trajectory, Deep Q-network (DQN), Engineering, Electrical and electronic engineering

## Abstract

Terahertz (THz) wireless communication is a promising technology that will enable ultra-high data rates, and very low latency for future wireless communications. Intelligent Reconfigurable Surfaces (IRS) aiding Unmanned Aerial Vehicle (UAV) are two essential technologies that play a pivotal role in balancing the demands of Sixth-Generation (6G) wireless networks. In practical scenarios, mission completion time and energy consumption serve as crucial benchmarks for assessing the efficiency of UAV-IRS enabled THz communication. Achieving swift mission completion requires UAV-IRS to fly at maximum speed above the ground users it serves. However, this results in higher energy consumption. To address the challenge, this paper studies UAV-IRS trajectory planning problems in THz networks. The problem is formulated as an optimization problem aiming to minimize UAVs-IRS mission completion time by optimizing the UAV-IRS trajectory, considering the energy consumption constraint for UAVs-IRS. The proposed optimization algorithm, with low complexity, is well-suited for applications in THz communication networks. This problem is a non-convex, optimization problem that is NP-hard and presents challenges for conventional optimization techniques. To overcome this, we proposed a Deep Q-Network (DQN) reinforcement learning algorithm to enhance performance. Simulation results show that our proposed algorithm achieves performance compared to benchmark schemes.

## Introduction

Terahertz (THz) communications have been regarded as a promising candidate for future wireless communication systems, to support the explosive growth of mobile devices, with ultra-high bandwidth and minimum time delay, reliability, and seamless multimedia applications for 6G wireless networks^1–4^. THz communication spectrum range (0.1–10 THz) to assure a low-latency and dependable exchange for data rate by utilizing its very high data rate with the high quality of service when properly deployed to satisfy requirements of ultra-broadband in future wireless communications^5,6^.THz network supports emerging applications such as touchless mobile technologies, online gaming, videos with high-quality streaming, virtual reality (VR), augmented reality (AR) and similar applications necessitating data rates of up to 10 Tbps^7,8^. THz technology holds the potential to deliver data rates of up to 1 Tbps and can reduce the latency delay which can offer a promising solution to meet these requirements. However, THz communications have the advantage of increasing the bandwidth by orders of magnitude, but they inherently suffer from limited coverage due to the path loss incurred at high frequencies, the absorption of molecules in the atmospheric medium and the higher probability of line-of-sight (LOS) blockage. Thus, could lead to effective integration of wireless networks might be accomplished, by using THz-capable flying platforms in the communication network, such as Unmanned Aerial Vehicles (UAVs). UAV primarily perform communication functions at the air base network level of THz band in 6G^9^. UAVs can be utilized to assist communication and accomplish network extension at the wireless communication network level, hence improving the quality of wireless communication networks^10,11^. Furthermore, 6G will empower ultra-reliable and low latency communications (URLLC), enabling a wide array of applications in mobile edge computing (MEC) systems, such as UAV enabled relaying for MEC systems for transmissions in 6G networks^12^. In addition, researches direction on designing an algorithm for UAVs and positioning for multiple-user communication in the field of environment monitoring and applied in industry and military^13^. Because of limited energy supply for UAV, energy consumption has been one of the primary challenges of UAV in wireless communication, and how it can provide seamless and long-term coverage for this is still an open problem^14,15^. Recently, an energy-efficient communication method called Intelligent Reconfigurable Surface (IRS) has been introduced to enhance radio propagation capability. Recent researches that aim at the implementation of IRS in THz communications to evaluate its potential for enhancing coverage and achieving higher data rates and reducing time^16,17^. IRS that is made up of an array of reflecting elements, and each element enhances the quality of the initial received signal. With regard to 6G wireless communication THz networks, IRS can establish a connection with an airborne UAV to realize a practical way to support heterogeneous customers from the sky^18^. The results of this interaction in the creation of new an IRS class known as a flying IRS. It is consisting of an airborne vehicle that transports the IRS, which carrying IRS as a reflector. This makes it possible to deploy the IRS in a flexible manner and to reflect 360° in all directions, which can enhance the THz communication systems area coverage extension. In addition, increasing IRS deployment in the system is less expensive than adding more base stations (BSs).^19,20^. Thus, system performances of integrated UAV-IRS in THz networks, including service time and energy consumption for UAVs, depend on relay deployment and selection as well as THz band, which is extremely challenging for the networks. Researchers concentrate on optimizing UAV trajectory to minimize mission completion time of UAV. In^21^, the hovering location and the trajectory of UAV were studied. Number of UAVs, total mission completion time were kept for a minimum under the constraints for UAV energy budget and data transmission of IoT equipment. Authors in^22^ divided the problem into applied the successive convex approximation technique and a discretized equivalent. They optimized the performance of UAVs to communicate using terahertz frequencies, to minimize the all-UAVs mission completion time. In^23^, UAV not only needed for completing missions from users, also it reduces the overall flight time utilizing dynamic programming approaches and bisection search, to improve energy efficiency of UAV under certain UAV energy storage. The authors in^24^ aimed to minimize UAV mission time based on UAV’s energy budget by using UAV to disseminate information to devices in the communication system. Through the minimization of the weighted energy consumption for UAV, the tradeoff problem between the energy consumption of the UAV and all terrestrial devices was studied in wireless networks^25^. The authors in^26^, studied how can the UAVs support THz communications and the IRS was implemented to help the transmission. The objective is to optimized and evaluated the mission time optimization problem for terahertz UAVs with the goal of maximizing the minimal average rates of all users. The authors of^27^ employed Deep Reinforcement Learning (DRL) to study the problem of UAV trajectory in THz communication networks. They considered the throughput and trajectory maximizing UAV while reducing UAV transmit power. In^28^ the authors consider a Federated learning (FL) network in IRS-assisted UAV communications, in order for minimize the worst case mean square error (MSE) by jointly optimizing the UAV trajectory the IRS phase shift, and the user’s transmission power. The authors in^29^ jointly optimized flight trajectory of UAV and transmission power for each user in THz assist UAV communication to minimize the total system delay, using DRL with primal proximal policy algorithm. In several recent studies, UAV-IRS systems have been investigated, but most of the studies were conducted under THz communication system model with only a single UAV-IRS trajectory deployed to minimize completion time. They neglected to determine the appropriate number of UAVs-IRS set while designing UAV-IRS trajectory in THz network. Due to the limitation of the UAV energy, a fixed number of UAVs-IRS may not be able to complete the mission. Given the above challenges, there are some issues need to be addressed before planning multi UAV-IRS trajectory enabled THz networks: one issue is how many UAVs-IRS should be dispatched in network to complete the mission time, considering energy limitation of UAV-IRS? Second issue is determination the optimal UAVs-IRS hovering points location to serve a user’s in the coverage area to optimize the trajectory of UAV-IRS? This makes it both essential and challenging to achieve a good tradeoff between network coverage and network efficiency.

To address the above challenges, the motivation of this work we formulate the problem of designing multi UAVs-IRS trajectory assist-THz network and focus on downlink communications while taking into account energy consumption constraint of the UAVs-IRS, hovering point location of UAVs-IRS to minimize completion of mission time of UAVs-IRS. However, in proposed work the total time mission completion time, including the flying time, hovering time, and operation time duration is viewed as a combined indication, and the results that follow highlight the benefits of proposed algorithm by finding optimal UAV-IRS trajectory while minimize the system energy consumption. The main contributions of proposed work are summarized as follows:

- UAVs-IRS trajectory design aided-THz wireless communication system, operating in the THz frequency band, where the direct path between BS-UEs is blocked is considered in this study.
- A framework optimizing UAVs-IRS trajectory is introduced, aiming to minimize UAVs-IRS mission completion time, taking into account energy consumption constraint of the UAVs-IRS.
- A low complexity Deep Q-network (DQN) reinforcement learning based algorithm is proposed to finds an optimal low mission completion time and total energy consumption under various practical constraints such as flying time, hovering time, and the UAVs-IRS coverage area.
- Simulation results demonstrate effectiveness of proposed algorithm compared to benchmark schemes in terms of completion time of trajectory, with many actual parameter settings.

This paper introduces an optimization strategy aimed to find an optimal trajectories of UAVs-IRS such that minimized total mission completion time of UAVs-IRS while satisfying the energy consumption constraint. The system model of the proposed algorithm is detailed in “System Model Design” section. “Problem Formulation” section outlines the formulation of optimization problems, accompanied by the framework of the proposed algorithm. “Framework of the proposed algorithmm and policy” section provides the details of the proposed algorithm. Furthermore, “Time Complexity Analysis” section presents time complexity analysis proposed algorithm. “Simulation Results And Discussion” section discusses the results obtained. Finally, “Conclusion” section summarizes the paper.

## System model design

Initially, in this section, the network model, the channel model, the UAV-IRS model, the energy consumption model of UAV-IRS, the obstacle model and the area size decomposition are introduced.

### Network model

In this section, a downlink transmission UAVs-IRS aided THz wireless communication system is studied. We consider the downlink system model of a wireless network operating in the THz (0.3–10 THz) bands, to support outdoor applications, which consists of one BS operating in THz band that located at midpoint the urban area denoted by $A$. Also, number of Ground Users (GUs) are denoted by $N$ which are uniformly distributed in the coverage area. While number for UAVs-IRS is denoted as $M$. The position of UAV-IRS $j$ is dispatched from the midpoint area, and UAVs-IRS will hover for some time above each group of users at Hovering Points (HO), to cater the users within that group. We assume that $\text{G }=\left\{{\text{G}}_{1,}\dots . {,\text{G}}_{K}\right\}$ where $K$ is number of groups of users. It is assumed that direct links between BS and the GUs are obstructed by building or other obstacles environmental. The positions of the obstacles are assumed to be known, which is out of the scope of consideration in this paper. Communications between BS and GUs have been assisted by UAV-IRSs which are considered to be using Uniform Planar Array (UPA). The number of IRS reflective elements are $I={I}_{x} {I}_{y}$ where ${I}_{x}$ and ${I}_{y}$ are IRS reflecting elements numbers along the x-axis and y-axis respectively. Assume that rotary-wing UAV-IRS supports the flying hovering mode without taking acceleration and deceleration into account. Specifically, the UAV flies to the hovering points with a constant speed and hovers at these points with static status for servicing all users in groups.

Three-dimensional Cartesian coordinate system used to model UAV-IRS $j$ in THz network, considered the coordinate for BS can defined as ${q}_{B}=\left({x}^{\text{BS}}= 0,{y}^{\text{BS}}= 0,{h}^{\text{BS}}\right)$, where ${h}^{\text{BS}}$ is BS altitude and coordinates of user $N$ is ${q}_{n}=({x}_{n}^{\text{GU}},{y}_{n}^{\text{GU}},{h}_{n}^{\text{GU}})$, UAVs-IRS $j$ coordinates is ${q}_{j }=\left({x}_{j}^{\text{UAVIRS}}\left(t\right),{y}_{j}^{\text{UAVIRS}}\left(t\right),{h}_{j}^{\text{UAVIRS}}\right)$, respectively.

**Figure 1:**
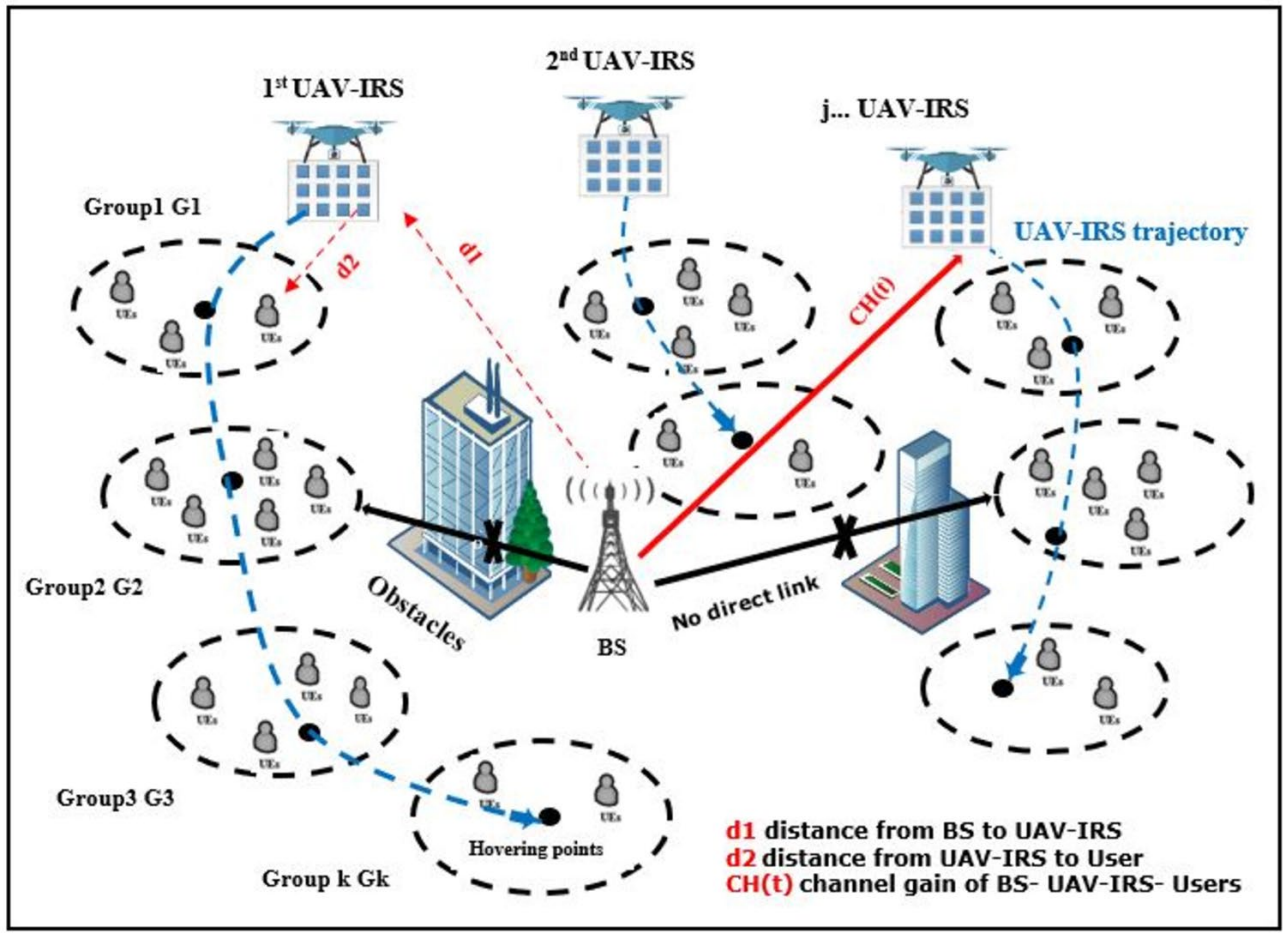
The illustration of system model.

The system architecture of UAV-IRS assisted THz wireless communication network is shown in Fig. 1. UAVs-IRS takes off from an initial point at the middle area ${\text{G}}_{1}$, determines the location for hovering point at Group 1 ${\text{G}}_{2}$ that will be visited first. UAV-IRS repeats this procedure until servicing all users for all groups is completed, and flies back to ${\text{G}}_{1}$.

### Channel model

As the key to understanding the THz band and laying out fundamentals for communications network, THz multipath channel model characteristics are essential for system design and performance evaluation. Channel models may include parameters such as sparse multipath loss, molecular absorption loss, diffuse scattering and specular reflection^30,31^. Consequently, channel transfer function is expressed as follows:

1 $$H\left( {f,d} \right) = \frac{c}{4\pi fd}e^{ - 0.5k\left( f \right)d}$$

where the term ${e}^{-0.5 k\left(f\right)d}$ indicates the channel path-loss due to the molecular absorption and the concentration of water vapor molecules in the air^32^, and $c$ is speed of light, $f$ is the carrier frequency. The $k\left(f\right)$ represents the absorption loss coefficient that is based on the transmission frequency and expressed as:

2 $$k\left(f\right)=\frac{P}{{P}_{\text{stp}}}\frac{{T}_{\text{stp}}}{T}{\sum }_{n,g}{Q}^{n,g}{\sigma }^{n,g}\left(f\right)$$

where $n,g$ is denoted the gases and all their isotopologue existing in air, respectively. The standard temperature is ${T}_{\text{stp}}$, ${P}_{\text{stp}}$ denotes the standard pressure, $T$ represents the temperature, and $P$ is the pressure. The transmission environment is indicating as ${Q}^{i,g}$, and ${\sigma }^{n,g}(f)$ represents the number of molecules per unit volume at frequency $f$^28^. Euclidean distance from BS to the UAV-IRS and from UAV-IRS to GUs represents as $d =$ ${d}_{1}+{d}_{2}$, where ${d}_{1}$ is distance between BS and first reflector of IRS, and ${d}_{2}$ is the distance between first reflector of IRS and GUs, can be expressed as follows:

3 $${d}_{1}=\sqrt{\left({x}^{\text{BS}}-{x}_{j}^{\text{UAVIRS}}\left(t\right)\right)+\left({y}^{\text{BS}}-{y}_{j}^{\text{UAVIRS}}\left(t\right)\right)+\left({h}^{\text{BS}}-{h}^{\text{UAVIRS} }\right)}$$

4 $${d}_{2}=\sqrt{\left({x}_{j}^{\text{UAVIRS}}\left(t\right)-{x}^{\text{GU} }\right)+\left({y}_{j}^{\text{UAVIRS}}\left(t\right)-{y}^{\text{GU} }\right)+\left({h}^{\text{UAVIRS}}-{h}^{\text{GU}}\right)}$$

### Coherence time

Coherence time is a temporal measure of the channel’s coherence. It is referring to measure of the time duration over which the wireless channel impulse response remains constant sufficiently for effective communication^33^. In the THz frequency band, due to the extremely high frequency and dynamic environments, the coherence time is typically very short. Particularly, we would like to clarify our system model can be acquired with the longer coherence time, which increases the accuracy of the distance determination. To manage coherence time effectively in system model to ensure reliable communication, we utilize a fixed user in multiple UAV-IRS scenarios in THz network offers several advantages including stability, typically experiencing more stable channel conditions since they are not moving relative to the network infrastructure.

### Transmission strategy

In the transmission strategy, UAV-IRS is used as the air relay to transmit information between BS and GUs, assuming that direct links are blocked due to blockage in the environment. The wireless signal is transmitted to the receiver through channel transfer function in THz band.

Additionally, the adaptability of UAVs-IRS allows them to relocate by sensing channel characteristics to improve actively channel conditions. Consequently, channel conditions are actively modified compared with fixed infrastructure communication. IRS is equipped with ${I}_{x} {I}_{y}$ elements of phase shift to establish the reflection communication link among BS and GUs through regulating IRS phase shift. Thus, the channel gain of BS—UAV-IRS—GUs can be represented by^34^:

5 $$G \left(\text{t}\right)=g \left(\text{t}\right) {e}_{t }\left(\text{t}\right) \Phi { e}_{r }(\text{t}{)}^{T}$$

where, $\Phi$ is beamforming matrix of IRS which is the diagonal phase shift matrix, defined as: $\Phi =diag[{w}_{1}{e}^{j{\phi }_{1}}, {{w}_{2} e}^{j{\phi }_{2}},\dots .. {{w}_{n} e}^{j{\phi }_{n}} ]$ where ${w}_{n}\in \left[0, 1\right]$ is an amplitude reflection coefficient of the reflective element of IRS, $\phi \in \left[0, 2\pi \right]$ is the phase shift of IRS element, and $j$ is a complex number's imaginary unit.

${e}_{t }\left(\text{t}\right)$ indicates the transmit vector from BS to UAV-IRS and ${e}_{r }(\text{t})$ is received vector from the UAV-IRS to GUs, respectively. Finally, the cascade channel of BS- UAV-IRS- GUs link as follows^35^:

6 $$g\left(t\right)=\frac{c}{8\sqrt{{\pi }^{3 } }f {r}_{t }\left(t\right) {r}_{o }\left(t\right)}{e}^{-j2\pi f \frac{\left( {r}_{t }\left(t\right)+{r}_{o }\left(t\right)\right)}{c}} {e}^{-\left(-0.5k\left(f\right)\left({r}_{t }\left(t\right)+{r}_{o }\left(t\right)\right)\right)}$$

where, ${r}_{t }\left(t\right)$ is the transmission vector array from BS to the first component of UAV-IRS, and ${r}_{o }\left(t\right)$ is the transmission vector array from first component of UAV-IRS to GUs.

### UAV-IRS model

Without loss of generality, we assumed UAVs-IRS $j$ fly with fixed altitude $h$, moving at a constant speed $v$ in (m/s) and not exceeding maximum speed ${v}_{\text{max}}$. At each time slot period ${\updelta }_{t}$, it can move with the maximum distance as ${d}_{\text{max}}={v}_{\text{max}}{ \delta }_{t}$*.* In each time slot $t$, UAV-IRS will move with flying action determined by an angle of ${\upmu }_{t}\in [\text{0,2}\pi$], and a distance of ${\text{d}}_{t}$ ∈ [0, ${\text{d}}_{max}$]. This work only addresses 2D trajectory optimization, as the environment is dominated by high-rise buildings that would be necessary long climbing phases to be overflown. Based on the movement of UAV-IRS, the position of UAV-IRS will change after each time slot $t$, and the moving distance will be calculated as follows:

7 $$x_{j}^{{{\text{UAVIRS}}}} \left( t \right) = x_{j}^{{{\text{UAVIRS}}}} \left( {t - 1} \right) + v\left( t \right) \delta_{t} \cos \varphi_{t}$$

8 $$y_{j}^{{{\text{UAVIRS}}}} \left( t \right) = y_{j}^{{{\text{UAVIRS}}}} \left( {t - 1} \right) + v\left( t \right) \delta_{t} \sin \varphi_{t}$$

where $\varphi_{t}$ is UAV-IRS direction at time slot $t$ from UAV-IRS $j$ to the users $n$. Also, we assumed that the trajectory of UAV-IRS $j$ can visit all sequence of locations of stop points ${\text{G}}_{j}$ in ${Q}_{j}({T}_{j}^{F})$ selected as:

9 $${Q}_{j} ( {T}_{j}^{F} ) = [ {x}_{j}^{\text{UAVIRS}}(t),{y}_{j}^{\text{UAVIRS}}(t),{h}_{j}^{\text{UAVIRS}}{]}^{T}$$

This paper assumes that the entire process of deployment UAVs-IRS is divide into T time slots of unequal length, and each time slot $t$ with sufficient slot length ${\updelta }_{t}$. For ease of exposition, we divide it into three components. So, the completion time of UAV-IRS trajectory is directly related to the summation of the flying time, the flying hovering, and operation time of UAV-IRS $j$. The overall completion time of UAV-IRS $j$ is as follows:

#### The first phase UAVs-IRS processing time ${T}_{j}^{pro}$

Based on the group's current location and avoiding obstacles, UAVs-IRS calculates the next optimal location, which includes the planning duration time, which varies depending on the complexity of the mission, and the trajectory execution (trajectories are designed based on the specific requirements of a mission, taking into account factors such as optimal UAV-IRS location, hovering points changes).

#### The second phase UAVs-IRS flying time ${T}_{j}^{F}$

Time required for UAV-IRS to fly from the present hovering point to the next new hovering point. In other words, we need to decide the visit order of the hovering point to minimize the flight distance of UAVs-IRS and thus minimize the flight time. Let ${\text{G}}_{1}$ and ${\text{G}}_{K}$ be the optimal starting and ending group positions of UAV-IRS respectively. Moreover, the order of visiting the hovering points is expressed as ${\text{G}}_{1},\dots .{\text{G}}_{k}$ and let the order set $\text{G}$ to present UAV-IRS whole trajectory be:

10 $$G = \left\{{\text{G}}_{1},\dots \dots .. {,\text{G}}_{k} \right\}$$

According to UAV-IRS speed $v$, it is possible to determine UAV-IRS flying Time ${T}_{j}^{F}$ which can be represented as:

11 $${T}_{j}^{F}=\sum_{i=1}^{\text{k}} \frac{d\left({\text{G}}_{i}-{\text{G}}_{j}\right)}{v}$$

where $d\left({\text{G}}_{i}-{\text{G}}_{j}\right)$ is the distance function that calculates the distance between hovering points ${\text{G}}_{i}$ and ${\text{G}}_{j}$.

#### The third phase UAVs-IRS hovering time ${T}_{j}^{hov}$

After UAVs-IRS selection of the hovering point of each group, and UAV-IRS visiting order to these hovering points. In this case, the hovering time of UAVs-IRS is the time it takes to associate with the users through communicating and collecting the data and serving users inside the groups.

Let ${T}_{j}^{hov}$ be the hovering time that the users in the k-th cluster communicate with UAV-IRS, which can be expressed by:

12 $${T}_{j}^{hov}=\frac{D}{{R}_{j}(t)}$$

where D is total data transmitted for users, and $R(t)$ is the achievable data rate for the downlink transmission from the UAVs-IRS $j$ to users $M$ at each time slot $t$ is^36^:

13 $${R}_{j}(t)={B}_{j}{\text{log}}_{2}\left(1+\frac{{\text{P}}_{\text{j}}*\text{CH}\left(t\right)}{\uppsi + {\text{B}}_{\text{j}} \left(\text{d }{\text{e}}^{-0.5 k\left(f\right)\text{ d }}\right){\upsigma }^{2}}\right)$$

where $\uppsi$ the network experiences interference, the channel gain is $\text{CH}\left(\text{t}\right)$, ${\text{P}}_{\text{j}}$ is transmit power from UAV-IRS to their GUs, ${B}_{j}$ is the total THz-bandwidth, and ${\upsigma }^{2}$ is the additive white Gaussian noise (AWGN).

Then, $k\left(f\right)$ the absorption loss coefficient, and the distance from BS—UAV-IRS—GUs represents $\text{d}$. The following total mission completion time for UAVs-IRS $j$ as follows:

14 $${T}_{j} ={{T}_{j}^{F}+T}_{j}^{hov}+{T}_{j}^{pro}$$

**Figure 2:**
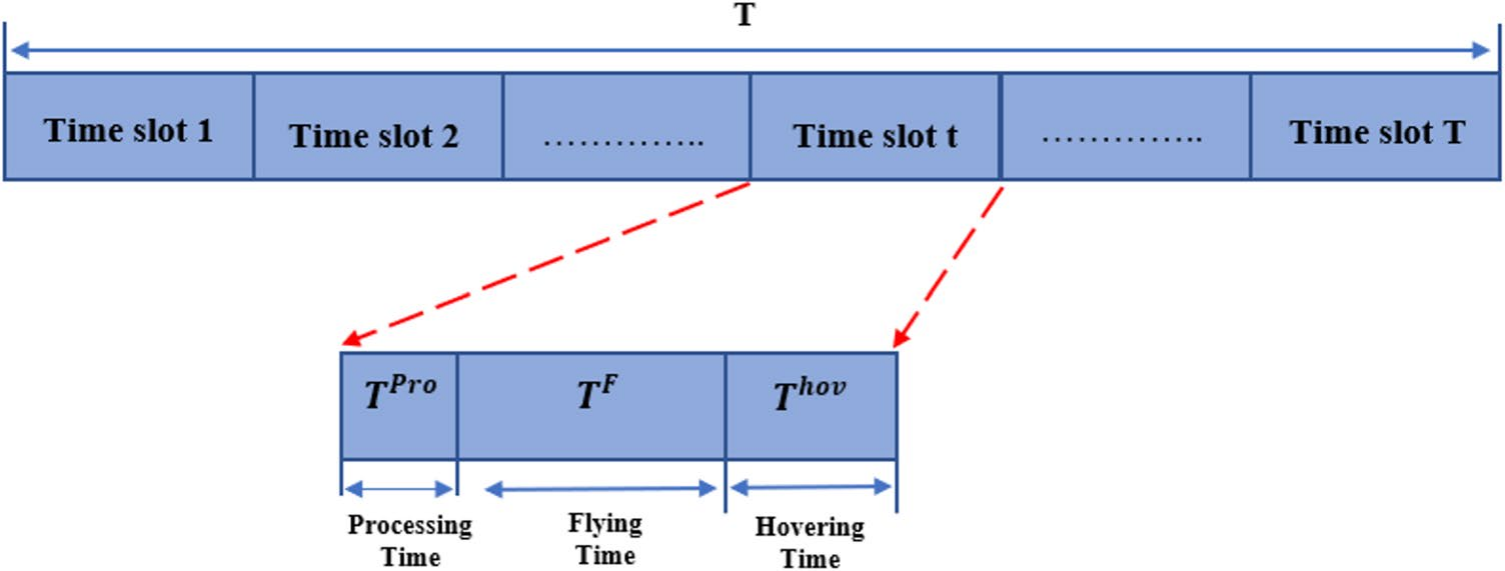
Diagram of time slot division.

The UAV-IRS responds to environment and makes decisions swiftly, so the processing and decision time is negligible compared to the flight time and hovering time of UAV-IRS, the division of time slots is displayed in Fig. 2 below.

### Energy consumption model

There are many factors that are involved of UAV-IRS energy consumption such as the energy during operations such as flying energy to keep UAV-IRS mobile and hovering energy. Regarding for the power consumption of the UAVs-IRS, it mostly consists of the communication-related power consumption and propulsion power consumption. The former primarily consists mostly of signal, circuitry, reception, and processing consumption, which is negligible compared with the propulsion power consumption. Therefore, the propulsion power consumption measures the energy consumed to fly or hover over UAVs-IRS. In general, energy consumption depends not only on UAVs-IRS velocity but also on its acceleration and deceleration. It should be noted that ignored the energy consumption during the acceleration and deceleration of UAVs-IRS^37^, which makes sense in reasonably where the acceleration and deceleration speed or acceleration/deceleration duration is minimal^38^. Therefore, power consumption of rotary-wing UAV-IRS flying with speed $v$ represents as:

15 $$P ={P}_{0}\left(1+\frac{3{v}^{3 }}{{U}^{3 }}\right)+{{P}_{1} }\sqrt{{1+\frac{{v}^{4 }}{4 {v}_{0}^{4}}-\frac{{v}^{2}}{4 {v}_{0}^{2}}} }+\frac{{d}_{0 }\rho s{v}^{3 }A}{2}$$

where ${P}_{0}$ and ${{P}_{1} }$ are blade profile power and induced hovering power state, respectively which are two constants linked to the physical parameters of the UAV-IRS. The tip speed of the rotor blade represents $U$, average rotor-induced velocity when hovering denotes ${v}_{0}$, the fuselage drag ratio is ${d}_{0}$, the rotor stiffness denotes as *s*, air density is ρ, and $A$ represents the rotor disk area. The relevant parameters are explained in details in Table 1. By substituting $v=0$ into (15), the hovering power consumption which is a finite value depending on the air density, aircraft weight, and rotor disc area, can be expressed as:

**Table 1:** Simulation parameters for system environment setting^26^.

Definition	Parameters	Values
Number of randomly distributed users	GUs	50–100
UAV-IRS altitude	${h}_{i}$	$50$ m
UAV-IRS flying speed	$v$	$10\text{ m}/\text{s}$
Carrier frequency	$f$	0.3 THz
Absorption coefficient	$k(f)$	0.005
Speed of light	c	$3\times {10}^{8}$ $\text{m}/\text{s}$
Bandwidth	$B$	0.1 THz
Separations between IRS reflectors	${\delta }_{x }{\delta }_{y}$	0.1m
Noise power spectral density	${\sigma }_{0}$	− 174 dBm/Hz
Transmit power of UAV	–	24 dBm
Tip speed of the rotor blade	$U$	200
Number of blades	b	4
Fuselage drag ratio	${d}_{0}$	0.30
Rotor solidity	$s$	0.05
Mean rotor induced velocity in hover	${v}_{0}$	7.2
Rotor disk area	*A*	0.79 ${\text{m}}^{2}$

16 $${P}_{h}={P}_{0}+{P}_{1}$$

As can be seen from (15) the propulsion power consumption of rotary-wing UAVs-IRS consists of three parts: induced, blade profile, and parasite power. The blade profile power and parasite power, increase cubically and quadratically, needed to control profile drag of blades and fuselage drag, respectively. However, induced power is needed to overcome the induced drag of the blades, that decreases with $v$.

After that, the total energy consumption of UAV-IRS used to determine the general consumption of energy denoted by $E$ during flight which is the sum of hovering energy ${E}_{j}^{hov}$ and flying energy ${E}_{j}^{F}$, and we deploy more than one UAV-IRS, so the fixed cost (e.g., take off or land in, maintenance) may incur ${E}_{j}^{pro}$.

Based on the above analyses, total energy consumption of proposed algorithm is expressed as:

17 $$E=\sum_{j=1}^{M}{{E}_{j}^{F}+E}_{j}^{hov}+ {E}_{j}^{pro}$$

wherein energy consumption of UAV-IRS flying ${E}_{j}^{F}$ given as follows: ${E}_{j}^{F}= {T}_{j}^{F}* {P}^{F}$ , where ${P}^{F}$ is the flight power of UAV-IRS during flight^14^, and ${T}_{j}^{F}$ is the amount of time the drone spent flying overall.

Moreover, ${E}_{j}^{hov}$ the energy consumption of hovering time is given as follows: ${E}_{j}^{hov}= {T}_{j}^{hov}* {P}^{hov}$, where ${P}^{hov}$ is the UAVs-IRS hovering power, and ${T}_{j}^{hov}$ hovering time, respectively.

### Obstacle model

In this paper, we assumed that the sizes and locations of the obstacles are known, since each blockage is represented as a rectangle with dimensions being the length, the width, and height of the obstacle, respectively. Obstacles can be measured by 3D point. In particular, we consider several obstacles, and their model are referred to in^39^. After that, GUs will be deployed randomly on the area size with these obstacles.

### Area decomposition

In this subsection, large area is decomposed depending on the available number of UAV-IRS. Aim to prevent UAVs-IRS from under-collision and avoid obstacles in environment, and balance the energy consumption for different UAVs-IRS. In order to achieve better coverage and improve the system's performance, the area is divided into various groups. As mentioned previously the number of UAVs-IRS is not predefined in the system. Inspired by the work in^37^, a modified k-means algorithm was used for divide users into various groups that are classified based on shortest distance criteria that allocate users to groups and can apply data rate constraints in accordance with predetermined requirements.

## Problem formulation

The objective function of this work is to find the optimal UAVs-IRS trajectory that minimizes the total mission completion time of UAVs-IRS while satisfying energy consumption constraints. Mission completion time includes the time taken by UAVs-IRS flying time, hovering time, and operation time. refers to the trajectory time taken by all UAVs-IRS to complete respective trajectory tasks. To maintain THz wireless communication system, our optimization problem can be formulated mathematically as a constrained optimization problem, where the objective function represents the total mission completion time of UAV-IRS and the constraints considering represent the energy consumption of UAVs-IRS that can improve the performance of the system. Regarding the optimizing trajectory problem, the UAVs-IRS seeks to find the optimal trajectory in minimum mission completion time is denoted as ${T}_{j}$. Therefore, the optimization problem can be formulated as the following:

18 $${\varvec{P}}1:\underset{{G}_{i},{q}_{j}}{\text{min}}\sum_{j=1}^{\text{M}} {T}_{j}$$

18a $${\varvec{s}}.{\varvec{t}}: \frac{d \left|({G}_{i}-{G}_{j} )\right|}{v} \le {V}_{max}$$

18b $$T\le \boldsymbol{ }{T}_{max}$$

18c $$E\le {E}_{max}$$

18d $$\left|{q}_{m}-{q}_{j} \right|\ge \text{Dmax}$$

18e $$0 \le {x}_{j}^{\text{UAVIRS}}\left(t\right)\le {x}_{max}$$

18f $$0 \le \boldsymbol{ }{y}_{j}^{\text{UAVIRS}}\left(t\right)\le {y}_{max}$$

18g $$0 \le \varphi_{t} \le 2\pi$$

where constraints (18a) indicate that the maximum distance travelled by the UAV-IRS $j$ cannot exceed the maximum speed ${V}_{max}$. Constraints (18b) the total mission completion time of UAV-IRS ${T}_{j}$ cannot be greater than ${T}_{max}$. Constraint (18c) indicates the transmission energy of the UAV-IRS has to be less than the maximum available energy. Constraint (18d) denotes that to keep a minimum secure distance of any two UAVs-IRS (to satisfy the collision avoidance. (18g) specifies that the movement direction of UAV-IRS between (0, 2π). While constructing UAVs-IRS trajectories ${Q}_{j}( {T}_{j}^{F})$ we have to consider the deployment optimization of UAVs-IRS stop points, and the number of UAV-IRS to find optimal trajectory time of UAV-IRS with minimum energy consumption.

Moreover, $P1$ consists of different types of variables like integer decision variables (e.g., the number of UAVs-IRS $j$ and hovering points locations).

## Framework of the proposed algorıthm and policy

The proposed algorithms are described in detail in the following subsections. The problem formulation of the proposed UAV-IRS trajectory algorithm which is an NP-Hard problem. The objective is to cover the area by passing through all hovering point locations, and minimizing energy consumption. In order to handle this problem in a tractable manner, we proposed DQN based algorithm and an Iterative heuristic algorithm for UAV-IRS Trajectory planning.

This section, will explain this DQN algorithm and heuristic approach to solve the formulated problem.

### Deep Q-network (DQN) algorithm

This subsection introduces the proposed DQN algorithm and optimization policy framework. According to the above system model, we design the trajectory UAV-IRS assisted THz communication system as an environment. Finding the optimal solutions in the dynamic environment is an effective dynamic programming techniques to solve the sequential decision-making problems. According to DQN proposed UAV-IRS assumed as agent is employed for interacting with the environment and approximate an optimal action-value function for maximizing the accumulated rewards within a sequence of states^40^. One of the popular Reinforcement Learning methods that combines Q-learning and neural networks is called DQN. Furthermore, DQN method has ability to handle non-linear function approximation and high-dimensional state spaces in complex environments with various obstacles. In order to improve the training effect and convergence rate DQN-based algorithm, we proposed to apply the iterative updating process and the Experience Relay (ER) method to correlations with the target by matching the action-values to periodically updated target values. And it improves data distribution by removing the correlation between observation states and random batch selection of samples^41^.

#### DQN algorithm processing framework

In the DQN-based algorithm framework consists of state ${s}_{t}$, the action ${a}_{t}$, and the reward ${r}_{t}$, action selection by an agent UAVs-IRS is accompanied by exploration and exploitation to obtain the highest reward even though updating current states. We initialize evaluation network, target network, and experience replay memory ${\mathbb{C}}$ from Lines 1 to 2. In this paper, during every episode, with the state variable ${s}_{t}$ is first initialized. Then the agent uses $\varepsilon$-greedy policy find balance between exploitation and exploration, which introduces parameter $\varepsilon$ with a small value to generate and chooses the action ${a}_{t}$. Specifically, the agent randomly selects from *A* with probability 1- $\varepsilon$ or chooses at that has the maximum Q value and explores the environment with probability. UAVs-IRS energy consumption is determined using Eq. (17). The reward is then obtained using Eq. (21). In line 10 transition will be stored in experience replay memory. From Line 11 the learning process starts by selecting randomly sampling M transitions from memory to train evaluation network. Evaluation network parameter updated by Eq. (22). Finally, there are periodic updates to target network as well. Therefore, proposed DQN algorithm, which is described in details in Algorithm 1.

The remaining corresponding elements, the state, action, and rewards, are clearly explained as follows:

##### State space

The state of agent has: the location of UAV-IRS (stop point current coordinate), we describe state space of all agents’ states for each episode are defined as

19 $$s_{t} = \left\{ {x_{j}^{{{\text{UAVIRS}}}} \left( t \right), y_{j}^{{{\text{UAVIRS}}}} \left( t \right)} \right\}$$

##### Action space

In this model, according to the observed state ${s}_{t}(\text{t})$, the agent uses discrete actions space to learn from the environment that maximizes the Q value. The agent (UAV-IRS) choosing a proper flying direction and arrives the next location to obtain a better reward. For each taken action, we assume that the agent UAV-IRS moves with a constant speed and the same altitude. More specifically, we assume the possible actions for the agent UAV-IRS are containing eight directions {forward, backward, left, right, northeast, northwest, southeast, and southwest}. All UAVs-IRS take actions one after another in a sequential manner. A large set of actions might be taken into account in this work but for simplicity purposes and without loss of generality, we maintain the most necessary actions to choose best UAVs-IRS flying direction in order to get greater rewards based on the current state ${s}_{t}(\text{t})$ the next state ${s}_{t}\left(\text{t}+1\right)$ which is trained by DRL-DQN algorithms. Thus, the action space ${a}_{t}(\text{t})$ is given by:

20 $$a_{t} = \left\{ {\varphi_{t} \left( {\text{t}} \right),t\left( {\text{t}} \right)} \right\}$$

$$\varphi_{t} \left( t \right) = \left\{ {{\text{forward,}}\,\,\,{\text{backward,}}\,\,{\text{left,}}\,\,{\text{right,}}\,\,\,{\text{northeast,}}\,\,{\text{northwest,}}\,\,{\text{southeast}}\,\,{\text{and}}\,\,{\text{southwest}}} \right\}$$

To be specific, indicates UAV-IRS remains still. Where $\varphi_{t} \left( {\text{t}} \right)$ is the direction of the UAV-IRS at time slot $t$ from UAV-IRS $j$ to the users $m$ , $t\left(\text{t}\right)$ is the time that the UAV-IRS moving at time slot $t$, respectively.

##### Reward function

The reward function determines the objective of the DRL problem, which is depending on the state ${s}_{t}$ and action ${a}_{t}$. In addition, the objective function of P1 is to minimize the mission completion time of UAV-IRS trajectory, while the objective of DRL (DQN) is to take an appropriate action to maximize the reward in any state ${s}_{t}$. Any action taken by the agent must satisfy the constraints specified in (18). If these restrictions are not adhered to, a penalty value is applied to the reward function.

Thus, as the reward function, the agent will learn to maximize this value, which effectively minimizes the completion time is given by:

21 $${r}_{t}: - \sum_{j=1}^{M}{T}_{j}$$

Motivated by the work presented in^42^, we provide here DQN-based algorithm for UAVs-IRS trajectory optimization. In DQN, UAV-IRS is controlled by an agent to interact with environment. It is assumed that are two DNNs named evaluation network and target network. It should be noted that even if the evaluation network has same structure as target network, but extremely periodically updates. Initially, the agent receives the state from the environment and forwards it to the evaluation network, that generates Q-values Q(${s}_{t}$, ${a}_{t}$) relevant to each action. The action is generated using greedy policy based on the Q-values^43^. Subsequently, the environment provides reward. The action space contains 8 elements which are varying from (A0, A1, A2, A3, A4, A5, A6, A7). To be specific, A0 means UAV-IRS flies forward, A1 implies UAV-IRS flies backward, A2 stands for that UAV-IRA turns to the left, A3 signifies that UAV-IRS flies towards right, A4 means the UAV-IRS flies northwest, A5 represents the UAV-IRS flies northeast, A6 indicates the UAV-IRS files southwest, and A7 represents UAV-IRS files southeast.Then, the transition is stored into an experience replay memory and transition consists of $({s}_{t},{a}_{t},{r}_{t} , {s}_{t+1})$ The learning process procedure starts when there are sufficient transitions in experience replay memory. In order to train the evaluation network, M transitions are randomly sampled in a mini-batch. The loss function for the evaluation network updating can be determined given the maximal Q-values max Q(${s}_{t}$`, ${a}_{t}$`) from target network and the Q-values Q (${s}_{t}$, ${a}_{t}$) from evaluation network. This can be expressed as:

22 $${L}_{i }({\delta }_{i } )= {\mathbb{E}}_{s,a}[ ( {r}_{t}+ \text{max}Q({s}_{t}`, {a}_{t}`\left|{\delta }_{i-1 } \right)-Q ({s}_{t}, {a}_{t}\left|{\delta }_{i }\right) {)}^{2}]$$

where ${\delta }_{i }$ denotes parameter of DNN, and $i$ represents index of iterations.

**Figure:**
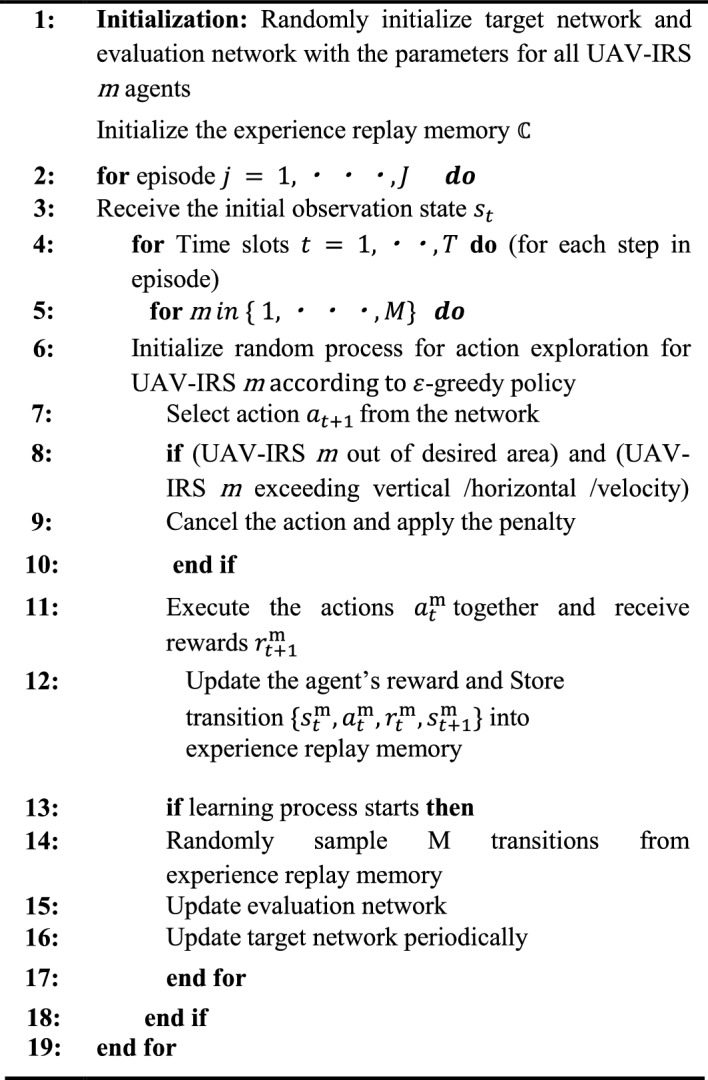
Algorithm 1: Proposed DQN algorithm for multi UAV-IRSs trajectory

### Iterative heuristic algorithm for UAV-IRS trajectory planning algorithm

In this section, the proposed iterative heuristic approach for UAVs-IRS trajectories algorithm framework is introduced. In order to optimize all UAVs-IRS trajectories, we proposed UAV-IRS trajectory optimization for Flying into Group ($\text{FIG}$), Hovering point ($\text{HO}$), and Flying out Group ($\text{FOG}$) in area of GUs.

Coordinates of passing points of UAV-IRS in the serving users in the area are expressed as:

23 $${\text{FIG}}_{j }\left( {\text{FIG}}_{{j}_{x} }, {\text{FIG}}_{{m}_{y} }, {\text{FIG}}_{{j}_{z} }\right)$$

24 $${\text{FOG}}_{j }\left( {\text{FOG}}_{{j}_{x} }, {\text{FOG}}_{{m}_{y} }, {\text{FOG}}_{{j}_{z} }\right)$$

25 $${\text{HO}}_{j }\left( {\text{HO}}_{{j}_{x} }, {\text{HO}}_{{j}_{y} }, {\text{HO}}_{{j}_{z} }\right)$$

Initially, from ${\text{FIG}}_{j}$ to ${\text{HO}}_{j}$ and ${\text{HO}}_{j}$ to ${\text{FOG}}_{j}$, UAVs-IRS fights to the hovering point ${\text{HO}}_{j}$ to serving all users inside Group. During the serving all users, the trajectory UAV-IRS flying from point ${\text{FIG}}_{j}$, passes through hovering point ${\text{HO}}_{j}$, and hovers at it for a while to serving all users inside Group, then flying out point ${\text{FOG}}_{j}$.

Based on the above analyses, we optimize UAV-IRS trajectory with the given UAV-IRS flying speed $v$. Flying time and hovering time of UAV-IRS affected by the $\text{FIG}$, $\text{FOG}$ and $\text{HO}$. Algorithm 2 describes overall architecture of the proposed iterative heuristic algorithm to minimize mission completion time of UAV-IRS.

The flying distance of UAV-IRS between ${\text{FOG}}_{j-1}$ and ${\text{FIG}}_{j}$ defined as ${dm}_{1}$. The shortest distance and longest distance between ${\text{FOG}}_{j-1}$ and ${\text{FIG}}_{j}$ represented as ${d}_{1min}$ and ${d}_{1max}$ respectively. Also, ${dm}_{2}$ denote flying distance of UAV-IRS between ${\text{FOG}}_{j}$ and ${\text{FIG}}_{j+1}$. Similarly, the shortest distance and longest distance between ${\text{FOG}}_{j}$ and ${\text{FIG}}_{j+1}$ are ${d}_{2min}$ and ${d}_{2max}$ , respectively. UAV-IRS needs to decelerate from the $\text{FIG}$ to $\text{HO}$ and accelerate from $\text{HO}$ to $\text{FOG}$, respectively.

Therefore, the total mission completion hovering time of UAV-IRS on the trajectory can be expressed as:

26 $${T}_{j}={t}_{{F}_{in}}\left({\text{HO}}_{j }\right)+{t}_{{F}_{ou}}\left({\text{HO}}_{j }\right)+\dots +{t}_{hov}\left({\text{HO}}_{j}\right)$$

where ${t}_{{F}_{in}}\left({\text{HO}}_{j}\right)$ indicates flying time of UAV-IRS from ${\text{FIG}}_{j}$ to ${\text{HO}}_{j}$, ${t}_{{F}_{ou}}\left({\text{HO}}_{j}\right)$ denotes flying time of UAV-IRS from ${\text{HO}}_{j}$ to ${\text{FOG}}_{j}$, and ${t}_{hov}\left({\text{HO}}_{j}\right)$ indicates hovering time of UAV-IRS at ${\text{HO}}_{j}$.

**Figure:**
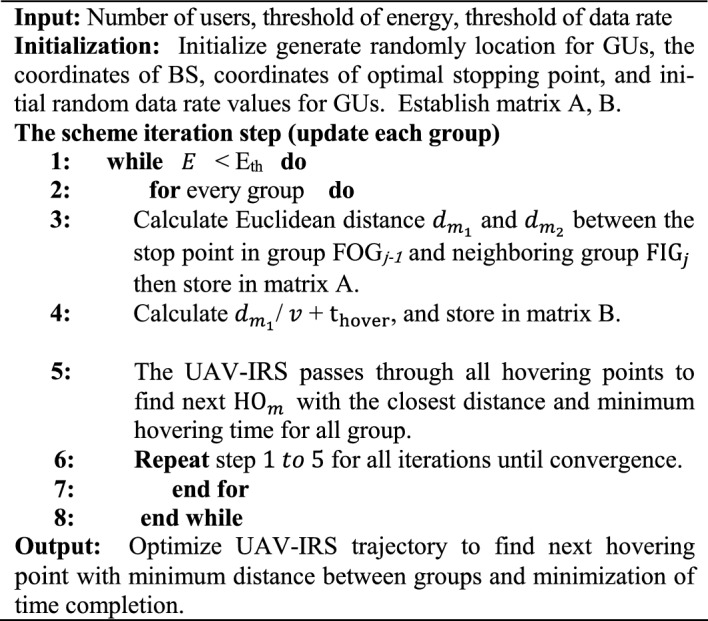
Algorithm 2: Heuristic Optimization Algorithm of UAV-IRS Trajectory

## Time complexity analysis

In this sub-section, we investigate the complexity of proposed framework, which is composed of DQN algorithm and the heuristic approach. The time complexity analysis is described as: Time complexity for heuristic approach is determined by O(K × I), where K represents the number of UAV-IRS stop points and I denote the maximum iteration numbers of the heuristic.

Time complexity of DQN algorithm: Algorithm 1 demonstrated after $\text{T}$ time slots and $\text{M}$ iteration rounds, DQN algorithm neural network parameters converge and tend to be stable. Number of operations of the network model is represented by the time complexity of a neural network and is determined by the number and layers for neurons in every layer of the network as well as the dimensions of the input state and action respectively. Number of layers of DQN neural network is expressed by $\text{L}$, and number of neurons in layer $l$ represented by ${u}_{l}$. Operation times of DQN neural network in each time slot can be represented by $\sum_{l=0}^{L} {u}_{l} {u}_{l+1}$. Thus, time complexity of DQN algorithm is $\text{O}(\text{ M T}\sum_{l=0}^{L} {u}_{l} {u}_{l+1})$.

## Simulation results and discussion

This section presents and verify the performance of proposed algorithm for outdoor environment scenario. Simulation was executed in Python programming language. Proposed DQN algorithm uses the same fully connected neural network structure which includes input layer for status information, an output layer that outputs the optimal action, two layers that are hidden in simulation using the function of activation, and modular normalization components. Additionally, to train policy network and Q-network using Adam Optimizers, and zero-mean normal distribution should be used to randomly initialize the values. It is important to note that simulation results shown were averaged over 500 independent iterations. Furthermore, we considering the urban scenario with area 50 m × 50 m, including one BS located in middle area which can serving 50 GUs. The locations of the GUs are uniformly distributed within the circle coverage area with radius r = 6 m, in each episode, the GUs locations assumed to be fixed. To avoid path loss peak, THz carrier frequency and THz transmission bandwidth operating in system model are set as $f$=0.3 THz and $B$ = 10 GHz, respectively. Other parameters are shown in Table 1^26^, and the settings parameters of network model are given in Table 2 according to^42^.

**Table 2:** Parameters settings for network model^42^.

Definition	Parameters	Values
Episodes number	$J$	3000
Each episode's number of steps	$T$	500
greedy policy	$\varepsilon$	0.9
Parameter of DNN	${\delta }_{i}$	0.99
experience replay memory	${\mathbb{C}}$	100,000
evaluation network with the rate		0.001
Optimizer		Adam

To evaluate the proposed UAVs-IRS trajectory algorithm performance, we compare and implement the following three benchmark schemes in a clear manner. Then we demonstrate the performance metrics: the mission completion time of UAV-IRS, the energy consumption, the average trajectory time, and the average throughput, for different UAV-IRS trajectory designs. All algorithms are deployed in the same environment for comparison and results are shown in the figures below:

Proposed algorithm: To evaluate how the proposed algorithm the optimal performance, we use DQN algorithm to get the optimal solution that can finds the optimal UAV-IRS trajectory with minimum mission completion time of UAV-IRS in THz network and minimize the energy consumption.

The Heuristic approach: In this scheme while searching for UAV-IRS trajectory, all feasible UAVs-IRS trajectories are listed and the optimal set of trajectories and usually solution is chosen the shortest trajectory to the target.

Random trajectory algorithm: This scheme is selected as an optimal UAV-IRS trajectory design, and each time UAV-IRS randomly selects the direction.

PPO algorithm: This algorithm is continuous action spaces, and typically find an iteratively trajectories using the current policy, estimating advantages for every state-action, and updating the policy parameters to maximize expected rewards while ensuring that the policy changes don't affect stability too much, to maximize expected rewards.

**Figure 3:**
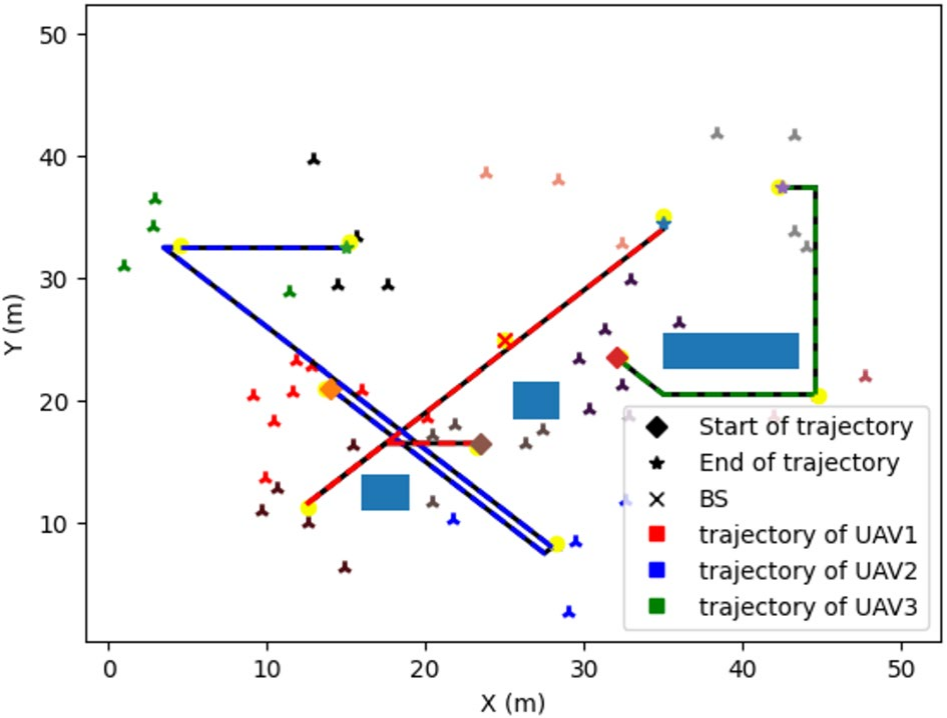
UAVs-IRS trajectory obtained by DQN. The illustration of system model. Each Group of users appears in Y-shape, and optimal location of UAV-IRS is yellow circle, respectively.

Figure 3, it is observed that UAV-IRS trajectory after training using the DQN algorithm, the model is trained on 10 groups of users in THz networks. Number of UAVs-IRS is three and UAV-IRS leaves from the initial location (25, 25). 50 users are random uniformly distribution in the area of size is at 50 m × 50 m. Additionally, we can observe that the Y-shaped marks of different colors represent the users which are distributed into different groups, the yellow of each circle represents the position of the optimal location of the group. The blue rectangles represent obstacles in simulation, and the line segments represent the paths taken by UAVs-IRS, the diamond-shaped represent the start of trajectory and the pentagrams the end of trajectory.

Finally, it is clear that no collision is detected with static obstacles is found throughout trajectories, and UAVs-IRS can weave effectively around obstacles. We utilize our improved DQN proposed algorithm to achieve the optimal trajectories for UAVs-IRS while minimizing the missions time of all UAV-IRS and minimum energy consumption. Hence, the final three trajectories of UAVs-IRS in this scenario model: the trajectory of UAV1-IRS represents red line {G0, G5, G4, G10}, the trajectory of UAV2-IRS represents blue line {G3, G6, G2, G1} and the trajectory of UAV3-IRS represent as green line {G7, G8, G9}.

**Figure 4:**
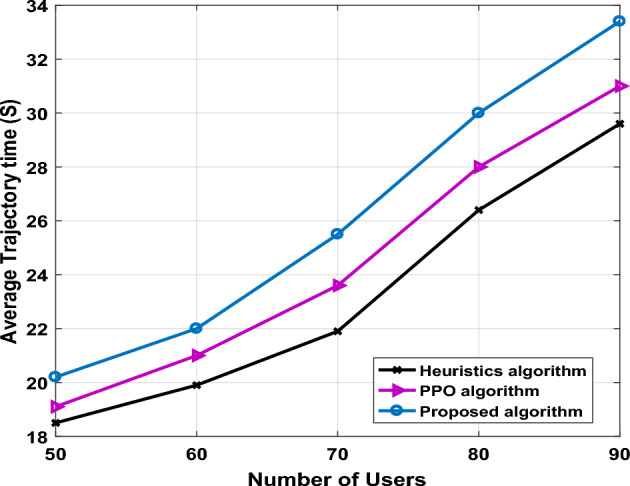
The average trajectory time versus number of users.

**Figure 5:**
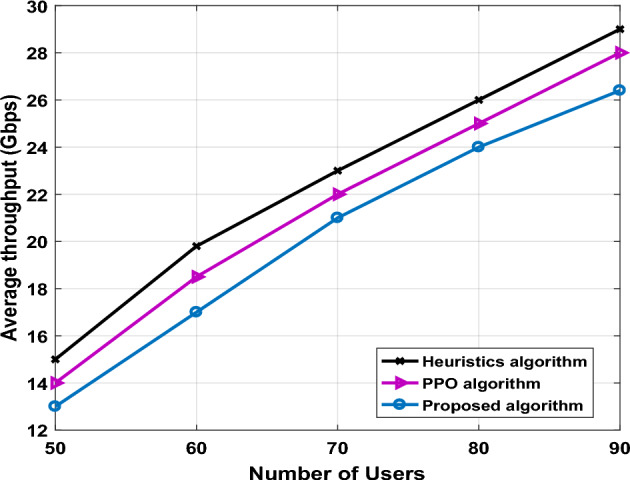
The average throughput versus number of users.

Figures 4 and 5 respectively show average UAV-IRS trajectory time (seconds) and the average throughput with different trajectory designs. As the number of users changes, the trend of average trajectory time is shown in Fig. 4. To evaluate performance of the different algorithms clearly, we define the average trajectory time as the summation of both flying time and hovering time, that can be given as:

27 $$T = T_{j}^{F} + T_{j}^{hov}$$

In this comparison, we utilize two benchmark algorithms as the comparison algorithms namely, heuristic algorithm based on the distance and PPO algorithm. It can be observed from Fig. 4 that proposed algorithm imposes the largest trajectory time as the increase of number of users. The PPO and heuristic algorithms consume much less UAV-IRS trajectory time compared with proposed algorithm. From practical aspects, the proposed algorithm is preferred with a relatively low complexity while achieving good performance. From practical aspects, the proposed algorithm is preferred with a relatively low complexity while achieving good performance. Nevertheless, the heuristic algorithm provides near-optimal performance and consumes less UAV-IRS trajectory time than the proposed algorithm, but the high computation complexity of the heuristic algorithm might limit its potential in practical scenarios. However, it can be observed that PPO algorithm may require more iterations to update its policy, especially in continuous domains that often learns more complex policies, to provides optimal converge solution.

Furthermore, we also compare the average throughput of proposed algorithm, heuristic algorithm, and PPO algorithm in Fig. 5.

### The average throughput

In our scenario, the average throughput is the summation of the UAV-IRS payload data per trajectory/ trajectory time in the combined scenario divided by the total number of UAVs-IRS, which can be determined as follows:

$${\text{The average throughput}} = \frac{{{\text{UAVs}} - {\text{IRS payload data per trajectory}}/{\text{ trajectory time }}}}{{{\text{total number of UAVs}} - {\text{IRS }}}}$$

Figure 5 evaluates the average throughput (Gbps) of all algorithms as a function of the number of users. We also present the case random trajectory optimization, PPO algorithm and heuristic algorithm for the comparison. Meanwhile, it has been observed in all schemes that increasing the number for users results in a higher overall average throughput. Specifically, the average throughput value of the proposed algorithm is very close to that of PPO algorithm. This is due to the fact that the PPO algorithm is continuous action spaces, that means it can handle the wide range actions and find all possible iteration (all available trajectories) to find an optimal solution without discretizing them. Furthermore, the heuristic algorithms assign the final path of UAV-IRS and always select the shortest path of UAV-IRS. This is normal and understandable in local path planning algorithms regardless of the increasing energy consumption than in our proposed algorithm designs. Another observation from Fig. 5 once number of GUs is 80, the proposed algorithm may achieve an average throughput of 24 Gbps. Therefore, when compared with another approaches, we can reach 26 Gbps as compared to heuristic algorithms, and by 25 Gbps at PPO algorithms. This is due to signal-to-noise ratio is significantly higher at THz frequencies because of the high path-loss characteristics of terahertz channels, leading to low interference among users. Although, performance of proposed algorithm is significantly inferior to that of heuristic algorithm, our computational complexity is $\text{O}(\text{ M T}\sum_{l=0}^{L} {u}_{l} {u}_{l+1})$, that is lower than heuristic approach. As a result, we achieved an appropriate trade-off between computational complexity and acceptable performance.

To investigate the effective mission completion time for UAV-IRS trajectories assisted THz network. It can be observed, that mission completion time defined as the trajectory time consumption of UAV-IRS and related to the flying time, hovering time, and processing time given in Eq. (28). Specifically, when UAV-IRS travelling from the first visited hovering point in a group to end optimal location in group, i.e., in completing its serving users task inside the area.

28 $${T}_{j} ={{T}_{j}^{F}+T}_{j}^{hov}+{T}_{j}^{pro}$$

**Figure 6:**
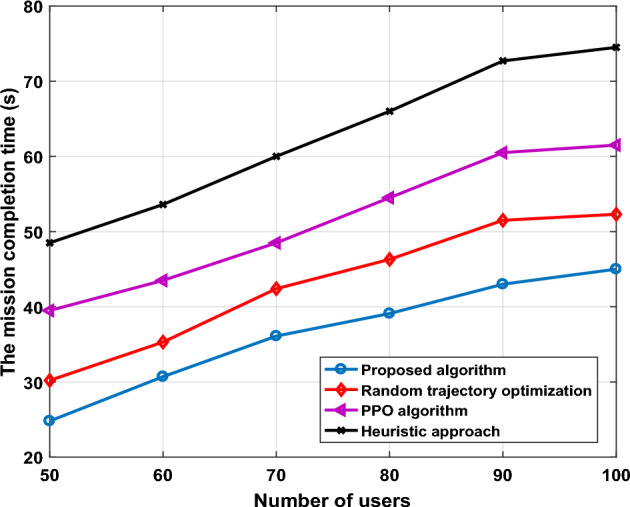
The mission completion time versus number of users.

In Fig. 6, we further compare the effective mission completion time of UAV-IRS for all algorithms, random trajectory optimization, PPO algorithm, and heuristic algorithm, for different values of users, when employing IRS with 64 elements, and set other parameters the same as those set in Fig. 5. It can be observed from the figure that with the increasing number of users, our proposed scheme achieves better results, the advantages of our scheme become more obvious, we optimized the trajectory of UAVs-IRS to minimize mission completion time of the system. This is expected since when we utilizing the location information for the optimal hovering points more wisely is beneficial for UAVs-IRS trajectory design, since it becomes time not wasteful for UAV-IRS to visit all the hovering points and find the optimal trajectory. When the number of users is large, the trajectory mission completion time is higher after PPO algorithm. This is because the agent UAV-IRS in PPO algorithm has to investigate a wider diverse of states and needs to explore more to find the optimal policies for each. This exploration may slow down the convergence towards the best solution, leading to longer mission completion time. However, random trajectory optimized is the high mission completion time, which can cause UAVs-IRS to waste a lot of time collection in transmission area. In addition, we considered the nearly passive IRS phase shift which adopts a combination of some passive reflecting elements and active reflecting elements in sequence, to reflect more signal paths, and elements of IRS to increase SINR at the cost of a slight performance loss.

**Figure 7:**
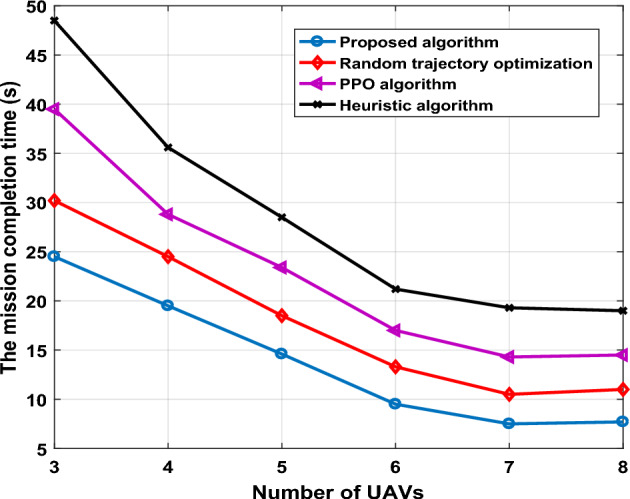
The mission completion time versus number of UAVs.

Additionally, we study the impact for different number of UAVs-IRS, on mission completion time, number of GUs was set 50 and we compared the other algorithms. Number of UAVs-IRS changes, mission completion time of UAVs-IRS is shown in Fig. 7. Obviously, increasing the number of UAVs could enhance the speed of monitoring area and serving of all users, and mission could be completed in less time. It can be seen from Fig. 7, when number of UAVs increases, mission completion time significantly reduces, compared with other algorithms our algorithm has achieved better results. This is because we consideration the energy constraint for each UAV-IRS in trajectory planning, and considers mission requirements to deploy an appropriate number of UAVs to complete the trajectory mission. However, increasing number of UAVs-IRS in the system may brought overhead, and reduce the energy efficiency.

**Figure 8:**
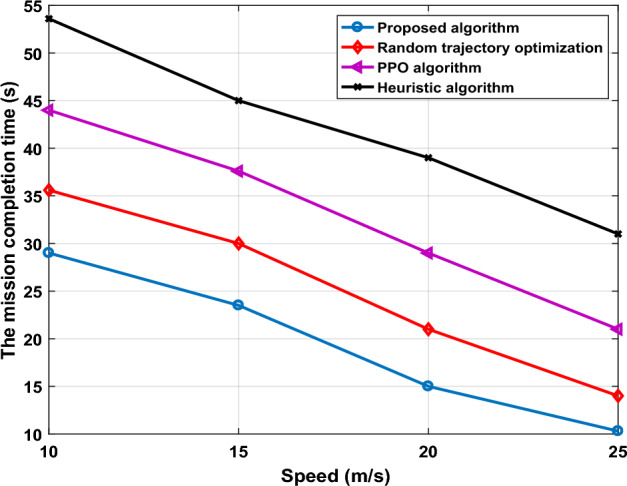
The mission completion time versus speed of UAVs-IRS.

Figure 8 shows the effect of flying speed $v$ on mission completion time performance when we set number of GUs are 60, 3 UAVs, area size set 50 m × 50 m and vary speed UAV-IRS from 10 to 25 m/s. It is observed time value decreases with the increases flying speed, i.e., serving more users can improve by increasing UAV-IRS flying speed, which comes from the fact that frequency of the information update increases as the speed increases in limited flying environment. Compared with benchmark scheme, time value decreases by 3.5%, 9.3% at $v$ =15 m/s and $v$ =25 m/s, respectively.

**Figure 9:**
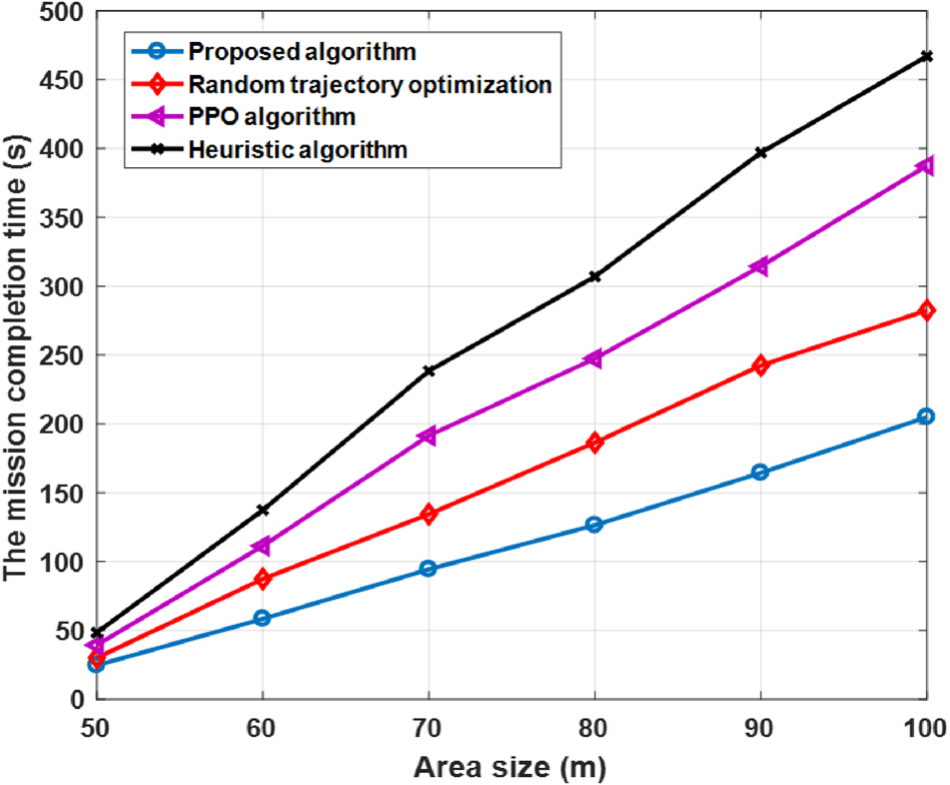
Different area size (m^2^).

Figure 9 illustrates mission completion time as the area sizes changes. Under different area size, we set number UAVs-IRS = 3 and the number for GUs at 55 and compared between algorithms. It was observed, that for larger network size area mission completion time was gradually increasing.

That was expected, since the larger area size caused a long flying time, UAVs-IRS could obtain more time to serve all users in the a large area size and the energy consumption is increased. We can observe that, when the mission area size is large, mission completion time of proposed algorithm is also lower than other algorithms. This is because the proposed solution in this paper allows UAVs-IRS to take full advantage of its advantages by decomposed the area is into various groups and choosing the optimal hovering points. Thus, UAV-IRS can fly less distance and serving all users more quickly, that allows UAV-IRS to have more energy to fly faster, to speed up the mission completion time at cost for consuming more energy of UAVs-IRS. We can observe, the highest mission completion time was achieved by the heuristic approach, where consumes a lot of energy. This is due to the high computation complexity of the approach might not adapt well to use in realistic scenarios.

**Figure 10:**
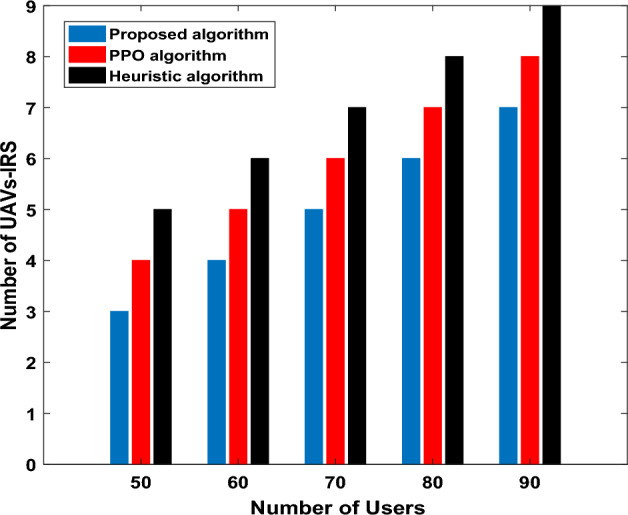
Number of UAVs comparison between different benchmark algorithms.

Figure 10, we test the proposed algorithm performance by evaluating number for UAVs-IRS versus the number of GUs. All results are derived by the same network configuration and results of more than 100 runs were averaged. Furthermore, number for UAVs-IRS required by all algorithms increases gradually with increasing number of GUs. This is due to the fact that more UAVs-IRS will be needed to provide wireless coverage for increasing the number of users (dispersed in multiple groups in the area). Proposed algorithm obtains better performance, we deployed UAVs-IRS in the optimal locations in the group to hover for some time where the user’s density is high. The selections are the best hovering locations in terms of the average distance of UAV-IRS to GUs to provide better service all users. Thus, we expect that proposed algorithm can reduces the required number of UAVs-IRS to serve GUs. This figure illustrates the significant savings in number of UAV-IRS with reduction of up to three UAVs-IRS for the scenario under evaluation when compared to alternative approaches.

### Energy consumption

Energy consumption is recognized as a crucial metric for performance evaluating for algorithms across various domains and applications. It illustrates how ability proposed approach can minimize energy consumption and ensure safety by avoiding collisions with obstacles.

**Figure 11:**
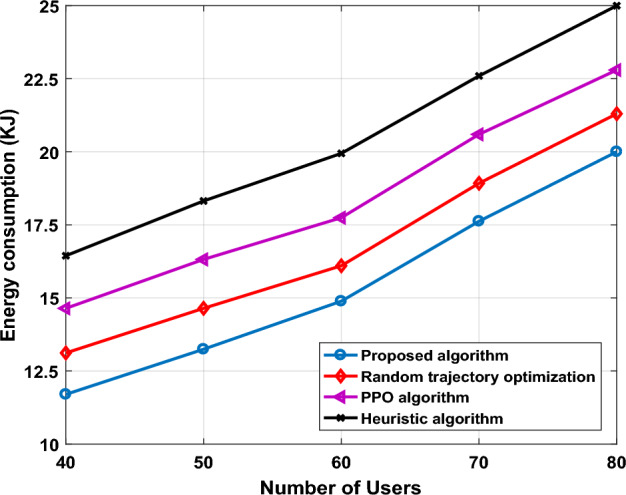
System energy consumption versus numbers of users.

In order to analyze the proposed algorithm that contributes to maximizing energy saving, energy consumption for proposed algorithm and benchmark schemes for various numbers of users is shown in Fig. 11. It can be easily inferred that energy consumption of the three methods goes up simultaneously with an increase in number of GUs, we find that proposed algorithm is always lower energy consumption than PPO algorithm. It can be observed that the heuristic approach illustrates the highest energy consumption, the reason is that heuristic approach obtains the solution by looking to finds an optimal trajectory, but it needs longer time to generate the solution and consume more energy. Therefore, the proposed algorithm will be achieved the best solution for finding the optimal trajectory for UAV-IRS with maximizes the energy saving of UAV-IRS. Thus, in proposed algorithm we using flying at maximum speed ${v}_{max}$ which can save more time to power more GUs, and can achieve the optimization goal of UAV-IRS trajectory to minimize total energy consumption.

Nonetheless, for complex system problems and the large scale, solution space is significantly exploding, result leading to inefficient application because of required time consumption.

### Energy-saving ratios

This metric quantifies the amount of energy saved relative to our proposed algorithm or benchmark algorithms. It is expressed as a ratio and indicates the effectiveness of an optimal trajectory planning in conserving energy. A higher energy-saving ratio signifies more efficient energy utilization. Therefore, the energy-saving ratios are calculated and Table 3 shows the results.

$${\text{Energy}} - {\text{Saving Ratios}} = \frac{{{\text{Benchmark algorithm }} - {\text{Proposed algorithm }}}}{{\text{Benchmark algorithm}}}{*}100$$

**Table 3:** Energy-saving ratios for the approaches when number of GUs increases from 40 to 90.

Users	Random trajectory optimaztion	PPO algorithm	Heuristics algorithm
40	12.1	25.1	40.5
50	10.5	23.2	38.3
60	8.9	22.4	36.3
70	7.3	16.8	28.2
80	6.5	13.9	24.9
90	6.7	14.5	24.1

Table 3 illustrates the the results of Energy-Saving Ratios for three approaches when number users increases from 40 to 90. As it is demonstrated, in the large scenarios where GUs is greater than 70 users, with expectations the proposed algorithm could achieve the optimal solutions that spend less time to server users and more energy saving than other benchmark schemes, that helps to reduce the effective energy consumption of UAV-IRS. This is due to proposed framework introduces DQN to carry out deployment optimization, which can find the optimal number of hovering points which improves efficiency of UAV-IRS trajectory planning. On the other hand, comparing PPO algorithm to DQN proposed algorithm in large scenarios where GUs is 90, the latter tends for provide better performance in Energy-Saving Ratio increasing through 6.7 and 14.5 respectively in PPO algorithm. Furthermore, proportion of energy consumption is relatively large in heuristic method.

**Figure 12:**
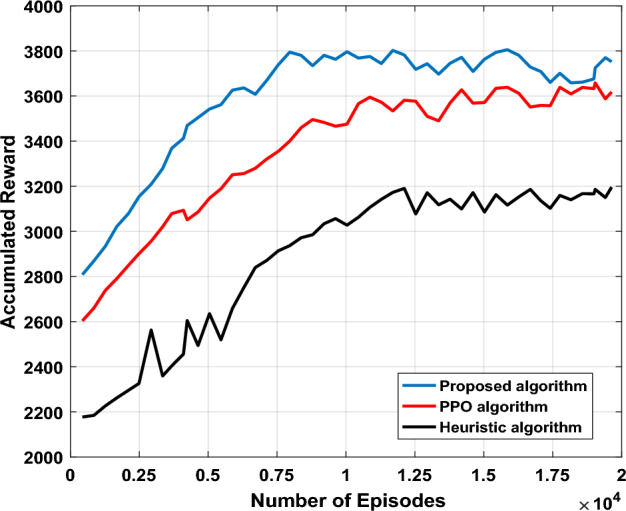
Accumulated reward under different algorithms.

Figure 12 shows the accumulated rewards of proposed algorithm, DQN, Heuristic approach and PPO algorithm. Initially, the proposed algorithm converges around 3700. PPO algorithm converges about 10,000 episodes, and the final reward value is 3600. Heuristic algorithm converges to 3100 around 12,500 episodes. Nonetheless, it can be seen that proposed algorithm achieves the largest average reward value and the fastest convergence speed, indicating the action selection strategy based on ε-pseudo count can more efficiently realize agent selection and utilization actions. Furthermore, performance gaps between proposed algorithm and benchmark algorithms are significant and keep increasing with iterations, that verifies proposed algorithm effectiveness.

**Figure 13:**
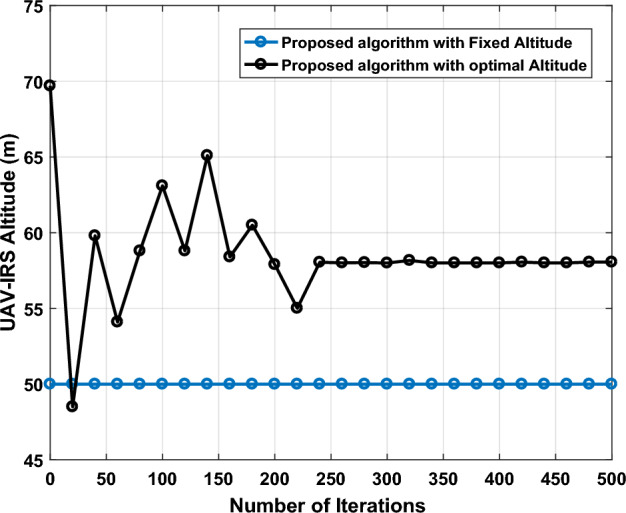
Proposed algorithm with fixed altitude and optimal altitude values in terms of Iterations.

Figure 13 shows the details of our proposed work by adjusting UV-IRS trajectory with fixed altitude and optimized altitude in terms of iterations. All results are derived by the same network configuration, we set number for GUs = 70, the altitude of UAV-IRS is constrained within the range from (50 m to 90 m). It can be seen from Fig. 13, when we used a constant altitude ${h}_{i}$=50 m which is very close to the obtained value of optimal altitude is ${h}_{i}$ = 58 m in 500 iterations. However, we find out that UAV-RS is adjusted to relatively low altitude. This is because the amount of the propulsion energy is not sufficient to attract the UAV-IRS to higher altitudes.

**Figure 14:**
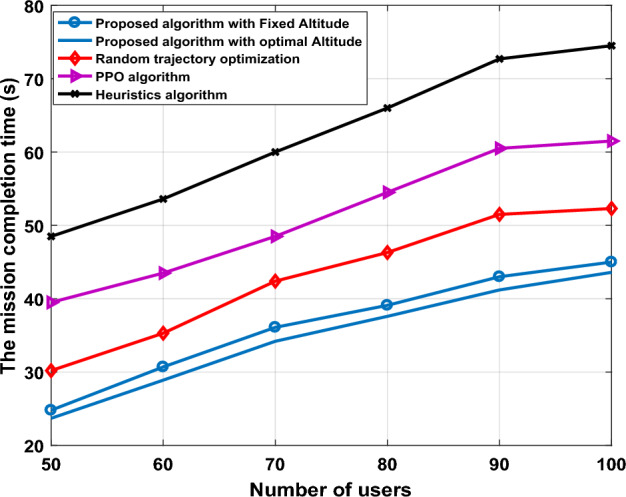
The mission completion time versus number of users with fixed altitude, and optimal altitude.

In Fig. 14, we further compare the effective mission completion time of UAV-IRS for all algorithms, random trajectory optimization, PPO algorithm, and heuristic algorithm, for different values of users, when utilized the fixed altitude and optimal altitude. The optimal altitude is automatically calculated by DQN proposed algorithm. After that, it is applied to UAV-IRS, and UAV-IRS is positioned at the calculated optimal altitude. According to the time of calculating the optimal height, UAV-IRS instantly positioned in the designated group at optimal altitude ${h}_{i}$ = 58 m. Finally, while planning and optimizing 3D trajectories into UAVs-IRS operations in THz network offers many benefits, like in terms of flexibility and can improved coverage. However, 3D trajectory of UAV-IRS in THz network presents additional issues compared to 2D scenario, due to characteristics propagation of THz band which is very sensitive to blockage and attenuation by obstacles, and can affect to maintaining reliable communication links with UAVs-IRS at varying altitudes, which requires more energy propulsion, and add more complexity. Thus, these factors need to be carefully considered and addressed in a more detailed manner in the future work, to ensure the optimal 3D trajectory of UAVs-IRS varying altitudes with low energy consumption in THz networks.

## Conclusion

This paper proposed framework for designing an optimal trajectory for UAVs-IRS algorithm assisted -THz wireless network. Accordingly, we proposed algorithm by optimizing trajectory of UAV-IRS, while ensuring UAV-IRS can successfully upload data rate from users inside groups with the remaining energy to minimize mission completion time for UAVs-IRS. Time minimization was formulated as a convex optimization problem. Consequently, to solve formulated problem, we proposed a low complexity approach to solving problem, Deep Q-Network (DQN) reinforcement learning algorithm which has advantage of training time, for improving performance. The algorithm finds the optimal time for mission completion of UAV-IRS trajectory to maximize the average throughput data rate of users, and minimize total energy consumption under various practical constraints such as flying time, hovering time and UAVs-IRS coverage area.

Simulation results revealed the proposed algorithm can achieved a good performance with significant increase in mission time compared to benchmark approaches scenario. In particular, proposed algorithm improves in mission time boosting the ratio by 60% greater than PPO algorithm and 93% compared with the heuristic approach. As a result, the effectiveness of optimal trajectory planning in conserving energy ratio in our proposed algorithm is up to 25% and 40.5% greater than PPO algorithm and heuristic approach, respectively. In addition, the results show that proposed framework significantly improving the convergence where it improved the accumulated rewards approximately 27% better than PPO algorithm. Future works, the static user scenarios are also a matter of concern, with limited applicability of the mobile user’s model in the real scenarios. We would like to extend our work in the next step by optimized 3D trajectory UAV-IRS considering the mobility of users and using appropriate artificial intelligence method to predict and generate real scenario that represents users’ mobility. Therefore, energy-efficient is a topic worth researching as well.

## Methods

To evaluate performance of proposed algorithm, extensive simulations are conducted. Simulation was performed in Python 3.7 and Tensorflow 1.15.0. In order to implement proposed DQN algorithm, we used AdamOptimizer that can update evaluation network with the rate 0.001. Also, we deploy two fully-connected hidden layers [256, 256] neurons and with 300 iterations target network updated. Mini-batch size, and experience replay memory are 10,0000 and 128 respectively. Every episode of the model training phase starts with initialized the task environment is randomly, that includes the locations of entities like UAVs-IRS, users, and obstacles that need to be avoided. Every episode is terminated when successfully finds the target with the highest reward, UAV-IRS runs out of battery UAV-IRS hits an obstacles.

## Data Availability

The datasets used and/or analyzed during the current study available from the corresponding author on reasonable request.
